# Implementation Intention and Reminder Effects on Behavior Change in a Mobile Health System: A Predictive Cognitive Model

**DOI:** 10.2196/jmir.8217

**Published:** 2017-11-30

**Authors:** Peter Pirolli, Shiwali Mohan, Anusha Venkatakrishnan, Les Nelson, Michael Silva, Aaron Springer

**Affiliations:** ^1^ Institute for Human and Machine Cognition Pensacola, FL United States; ^2^ Palo Alto Research Center Palo Alto, CA United States; ^3^ University of California, Santa Cruz Santa Cruz, CA United States

**Keywords:** mobile applications, models, theoretical, habits

## Abstract

**Background:**

Implementation intentions are mental representations of simple plans to translate goal intentions into behavior under specific conditions. Studies show implementation intentions can produce moderate to large improvements in behavioral goal achievement. Human associative memory mechanisms have been implicated in the processes by which implementation intentions produce effects. On the basis of the adaptive control of thought-rational (ACT-R) theory of cognition, we hypothesized that the strength of implementation intention effect could be manipulated in predictable ways using reminders delivered by a mobile health (mHealth) app.

**Objective:**

The aim of this experiment was to manipulate the effects of implementation intentions on daily behavioral goal success in ways predicted by the ACT-R theory concerning mHealth reminder scheduling.

**Methods:**

An incomplete factorial design was used in this mHealth study. All participants were asked to choose a healthy behavior goal associated with eat slowly, walking, or eating more vegetables and were asked to set implementation intentions. N=64 adult participants were in the study for 28 days. Participants were stratified by self-efficacy and assigned to one of two reminder conditions: reminders-presented versus reminders-absent. Self-efficacy and reminder conditions were crossed. Nested within the reminders-presented condition was a crossing of frequency of reminders sent (high, low) by distribution of reminders sent (distributed, massed). Participants in the low frequency condition got 7 reminders over 28 days; those in the high frequency condition were sent 14. Participants in the distributed conditions were sent reminders at uniform intervals. Participants in the massed distribution conditions were sent reminders in clusters.

**Results:**

There was a significant overall effect of reminders on achieving a daily behavioral goal (coefficient=2.018, standard error [SE]=0.572, odds ratio [OR]=7.52, 95% CI 0.9037-3.2594, *P*<.001). As predicted by ACT-R, using default theoretical parameters, there was an interaction of reminder frequency by distribution on daily goal success (coefficient=0.7994, SE=0.2215, OR=2.2242, 95% CI 0.3656-1.2341, *P*<.001). The total number of times a reminder was acknowledged as received by a participant had a marginal effect on daily goal success (coefficient=0.0694, SE=0.0410, OR=1.0717, 95% CI −0.01116 to 0.1505, *P*=.09), and the time since acknowledging receipt of a reminder was highly significant (coefficient=−0.0490, SE=0.0104, OR=0.9522, 95% CI −0.0700 to −0.2852], *P*<.001). A dual system ACT-R mathematical model was fit to individuals’ daily goal successes and reminder acknowledgments: a goal-striving system dependent on declarative memory plus a habit-forming system that acquires automatic procedures for performance of behavioral goals.

**Conclusions:**

Computational cognitive theory such as ACT-R can be used to make precise quantitative predictions concerning daily health behavior goal success in response to implementation intentions and the dosing schedules of reminders.

## Introduction

### Background

Mobile health (mHealth) systems provide new opportunities to provide precise individualized just-in-time interventions to support behavior change [1]. mHealth provides a path for translating evidence-based interventions (EBIs) onto delivery systems that are replicable, scalable, and sustainable, with great economies of scale for health care delivery [2]. mHealth also provides new opportunities to study and apply psychological theory in the ecology of everyday life, with a focus on meaningful health-related behavior [3,4].

In this paper, we present an exploratory 28-day study in which an mHealth app delivered interventions to support people in pursuing self-selected healthy behavior change goals such as eating more vegetables, eating more slowly, and increased walking time per day. The interventions included asking people to set implementation intentions [5]—an EBI that yields medium-to-large improvements in behavior change [6]—supplemented by reminders whose delivery schedule was experimentally manipulated to explore cognitive mechanisms that might amplify the effects of implementation intentions on behavior change. The adaptive control of thought-rational (ACT-R) theory of cognition [7] is used to develop an integrated mechanistic account of the role of implementation intentions in enhancing behavior change intentions and actions; how reminders have time-varying strengthening effects on the impact of implementation interventions; and how controlled, deliberative, goal-striving behavior becomes automatic and habitual. On the basis of this account, a predictive mathematical model is fit to the data from the mHealth study.

A key driver of health care costs in the United States and other developed nations are unhealthy but changeable behaviors such as physical inactivity and eating too much, or too much of the wrong things [8]. The working assumption for our own mHealth research is that to master the complex fabric of a new healthy lifestyle, one must master and weave together a new set of healthy habits that override the old unhealthy habits. mHealth platforms are proposed as systems that can expand and intensify psychosocial and health behavior interventions beyond clinical settings into the ecology of everyday life [9] to support the formation and maintenance of desirable healthy habits.

The study and model presented in this paper are part of a larger project (called Fittle+), with several aims. First, the project involves the iterative development of an integrated pervasive computing platform for delivering and testing multiple EBIs. Second, the Fittle+ project explores how specific EBIs in the literature—such as guided enacted mastery [10] and implementation intentions—can be translated to mHealth delivery. Third, the project pursues the development of an integrated psychological model of behavior change that encompasses multiple mechanisms and addresses the dynamic effects of the EBIs and other fine-grained mHealth interventions such as reminders.

In the rest of this introduction, we present:

- A summary of the theory of planned behavior (TPB).
- A review of implementation intentions as an EBI and the hypothesized underlying cognitive mechanisms of action for implementation intentions.
- A theoretical framework for long-term behavior change based on recent cognitive neuroscience that proposes dual systems: A system of mechanisms that supports volitional goal-striving and a system that supports habit formation and execution.
- ACT-R as a mechanistic and predictive dual-system theory of goal-striving and habit formation.
- ACT-R predictions regarding the effects of different reminder schedules on the strength of memory for implementation intentions.
- A dynamical mathematical model based on ACT-R that will be fit to individuals’ daily data over the course of the 28-day mHealth behavior-change study. The model addresses effects of implementation intentions, reminder schedules, and habit formation.

### Theory of Planned Behavior and Self-efficacy

The path to healthy habits is not simple. Much of the focus in behavior-change theory is on the factors that initiate and strengthen the intentional goal to change and the factors that strengthen the volitional and effortful striving to translate those goal intentions into actual behavior [11,12]. Despite criticism [13], a dominant theory of behavior change is the TPB [14]. The TPB proposes that volitional behavior change is a function of the *goal intention* to perform the behavior and *perceived behavioral control.* The goal intention is in turn a function of expectancy-value beliefs and attitudes. PBC is synonymous with the concept of *self-efficacy* in social cognitive theory [15,16] and predicts that the perception of the ease or difficulty of a particular intended behavior facilitates or impedes the intention to perform the behavior.

### Implementation Intentions

*Goal intentions* are hypothesized to be mental representations of desired behavior and end states, which are to be distinguished from *implementation intentions* that are mental representations of simple plans to translate goal intentions into behavior under specific conditions [5,17]. Interventions designed to foster the setting of implementation intentions typically ask people to specify when, where, how, and (sometimes) with whom to act on a goal intention by using if-then statements of the form: “If I encounter situation *S* then I will initiate action *A.*” It is argued [18] that one reason to focus intervention efforts on implementation intentions rather than goal intentions is that medium-to-large changes in commitment to goal intentions (*d*=0.66) only lead to small-to-medium changes in behavior (*d*=0.33) [19], but implementation intentions have medium-to-large effects on goal attainment (*d*=0.65) [6].

Wieber et al [18] review the experimental literature and studies of physiological correlates to bolster the hypothesis that two processes are involved in the effectiveness of implementation intentions: (1) the mental representation of situations in which the intended behavior is to take place becomes more accessible and activates the goal intention and (2) a strong associative link between a mental representation of the situation and intended behavioral action effects a heightened readiness to perform the action and the action takes less effort.

Previous research has manipulated the situation-action associative strength of implementation intentions and their effects on behavior using priming [20]. This implicates declarative memory processes [7,21] in mediating the effects of implementation intentions. This would also suggest that other ways of strengthening the declarative memory representations of implementation intentions should enhance their effectiveness—such as the explicit use of reminders to use implementation intentions. Prestwitch et al [22] presented a mHealth study that showed that SMS text message (short message service, SMS) reminders of implementation intentions promoted increased brisk walking but did not experimentally explore the specific effects of reminders.

### From Volitional Goal-Striving to Habits

Habits are only gradually learned through the association of specific behaviors to triggering cues in the environment, including physical settings and previous actions. More than a hundred years of psychological research on habits suggests that there are dual systems involved in habit acquisition and strengthening [23-25]. There is (1) a deliberative or controlled goal-striving process that motivates and guides the behavior in the relevant contexts and, through repetition (2) a habit is formed that is automatically performed without effortful, controlled goal-striving. Well-practiced habits appear to be performed automatically without mediating goals, motivation, or deliberative thought (system 1) [26], but habit formation typically depends on a long period of goal-mediated, consciously controlled, exploration, repetition, and practice of behavior (system 2) [26]. A simple example of this transition is developing the habit of keeping a food diary [27]. At first, one may need to set up reminders to go through the behaviors involved in recording meals, but eventually, the behavior can become triggered somewhat automatically at the end of every meal.

Research [25] suggests that the neural circuitry directing behavior undergoes changes as habits are acquired and strengthened. As new behavior is attempted, explored, and practiced, the prefrontal cortex communicates with the striatum (basal ganglia), and the striatum communicates with the midbrain and dopaminergic mechanisms aid learning and assign value to goals. Continued practice of the behavior forms a feedback loop between the sensorimotor cortex and the striatum, creating behavioral routines that appear to be units residing in the striatum. Habit learning is consistent with the learning of other (procedural) cognitive skills [25,28].

The underlying neurological mechanisms of habit learning are consistent with computational models of reinforcement learning [25] such as temporal-difference models [29] and the Rescorla-Wagner model [7,30,31]. Learning new sequences or organizations of behavior involves learning through experience the immediate value of actions that are currently available and the estimated value of future actions and basing choices on those learned value estimations.

### An ACT-R Model of Implementation Intentions, Reminders, and Habit Learning

In recent years, there has been push to develop rich, fine-grained, dynamical theories that are up to the task of predicting mHealth cause-effect relations and guiding the engineering of new personalized interventions [32]. Computational predictive models of self-efficacy (or PBC) have been developed based on dynamical control principles [33] and on cognitive theory [34]. In this paper, we extend the model of Pirolli [35] to provide a computational account of the mechanisms involved in *intention-to-behavior* processes [17] that are hypothesized to be improved by implementation intentions, reminders, and habit learning. The model presented here is based on the ACT-R theory [7], including recent extensions [36,37].

As was summarized in Pirolli [35], ACT-R [7,21] is a unified theory of how the structure and dynamics of the brain give rise to the functioning of the mind. The ACT-R simulation environment is a computational architecture that supports the development of models.

#### Modules and Buffers

ACT-R is composed of *modules*, processing different kinds of content, which are coordinated through a centralized *production module*. Each module corresponds to a brain region. Each module is assumed to access and deposit information into *buffers* associated with the module, and the central production module can only respond to the contents of the buffers. The *declarative memory module* stores memories of the kind of knowledge and experience that a person can attend to, reflect upon, and usually articulate in some way (eg, by declaring it verbally or by gesture). A consciously formulated implementation intention that is later remembered and acted upon is an example of something stored in the declarative memory module. The production module stores the habits and skills we display in our behavior without conscious awareness. The *goal buffer* stores and retrieves information that represents the internal intention of the system and provides local coherence to behavior. More specifically, the modules and buffers relevant to this paper include

- *Goal buffer* (dorsolateral prefrontal cortex), which keeps track of active goals and internal state of the system.
- *Production module* (basal ganglia), which matches the contents of other module buffers and coordinates their activity. The production module stores *production rules*. A production rule is a formal specification of the *flow of information* from buffers in the cortex to the basal ganglia and back again. As suggested by the literature review above, the production module is where new habits are stored. Productions have a *utility* property that is used to select the single rule that is executed.
- *Declarative module* (temporal lobe; hippocampus) and *retrieval buffer* associated with the retrieval of knowledge and past experiences from long-term declarative memory. The declarative module is where goal intentions are stored (before they become active goals in the goal buffer) and where implementation intentions are stored.

Knowledge in the declarative module and goal buffer is represented formally in terms of *chunks* [38,39]. Chunks have *activation* levels that determine the probability and time course of chunk retrieval into a buffer. Production utilities and chunk activations are real-valued quantities produced by *subsymbolic mechanisms* in ACT-R. These subsymbolic mechanisms reflect neural-like processes that determine the time course and probability of cognitive activity and behavioral performance. The dynamics of declarative memory retrieval and production selection are determined by these subsymbolic mechanisms.

#### Key Components of the ACT-R Model of Intentions, Reminders, and Habits

Pirolli [35] presented an ACT-R model motivated by Tobias [40] that is modified slightly here to suit the current experiment. The model includes the following components:

- *Goal intentions*: a goal-like representation that is stored in declarative memory as a kind of prospective memory [41,42] to be turned into an active goal, in the goal buffer, in response to the right context
- *Implementation intentions:* plan-like representations that are also stored in declarative memory to be turned into concrete behaviors by production rules
- *Reminders*: SMS text messages that cue the recall of implementation intentions and thereby, increase the activation of implementation intentions through learning mechanisms so that they are more likely to be retrieved in the right context in the future
- *Habit compilation (ie, production compilation)*: repeated execution of complex sequences of cognitive and behavioral steps (multiple production rules, multiple memory retrievals) produce new, simpler production rules that require less cognitive effort the next time around
- *Utility learning*: new habits are rewarded and slowly come to dominate over the old habits

#### Mechanisms Underlying the Dynamics of Reminders and Memory

**Figure 1:**
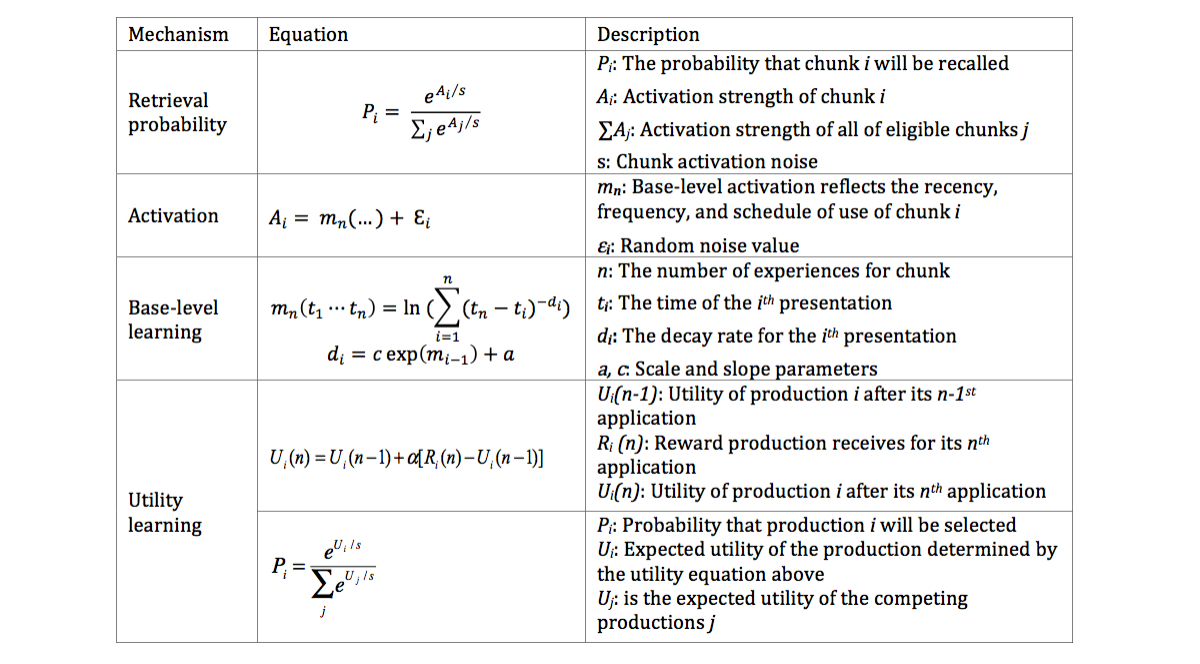
Some key adaptive control of thought-rational (ACT-R) subsymbolic mechanisms.

Figure 1 presents a subset of the ACT-R mechanisms relevant to the current model. The first two equations in Figure 1 define how the level of activation of chunks in declarative memory relates to the probability of their retrieval at any given time. The third equation for *base-level learning* defines how activation levels are increased by repeated experiences (practice) or decay with time (forgetting). These activation and base-level learning mechanisms are crucial to the ACT-R model of implementation intentions and the effects of reminders. Reminders for implementation intentions will increase their activation in declarative memory, but activation will decay as time goes by since those reminders were attended.

We hypothesize that the base-level strength of implementation intentions in declarative memory will be associated with improved success in achieving behavior-change goals. The dynamics of base-level strength will be related to frequency and timing of reminders, as well as the frequency and timing of actual use of the implementation intentions in performing behavior.

The base-level learning mechanisms defined in Figure 1 propose that each time an implementation intention is formulated, reminded, or put into practice, it receives an increment of activation (a practice effect). However, each increment of activation decays as a power function of time (the forgetting effect).

The rate of decay of each increment of activation depends on the strength of activation at the time of the reminding or practice: at longer intervals between remindings or practice, the activation levels are lower, and subsequent forgetting occurs less quickly (the spacing effect). When reminding or practice is spaced closely, the forgetting occurs more quickly.

#### Habit Learning Mechanisms

The last two equations in Figure 1 define utility learning and the relation of utility to the probabilistic choice of production rules to execute. These utility mechanisms are crucial to the ACT-R model of habit formation.

Also important in the ACT-R model of habit formation is the mechanism of *production compilation* [7,43,44] by which new production rules are acquired. A new production rule is generated every time two production rules are executed in sequence. The mechanism works to create new rules that eliminate internal cognitive processing, such as the need to retrieve information from the declarative module or set and maintain sequences of goals. Production compilation is viewed as the mechanism underlying the acquisition of new habits. Utility learning is a variety of reinforcement learning similar to temporal-difference learning [29] and Rescorla-Wagner learning [31]. According to the utility learning equation, the utility of a new production rule (habit) is gradually adjusted until it matches the average reward for using the production.

### ACT-R Predictions About Reminding Schedules and the Memory Strength of Implementation Intentions

**Figure 2:**
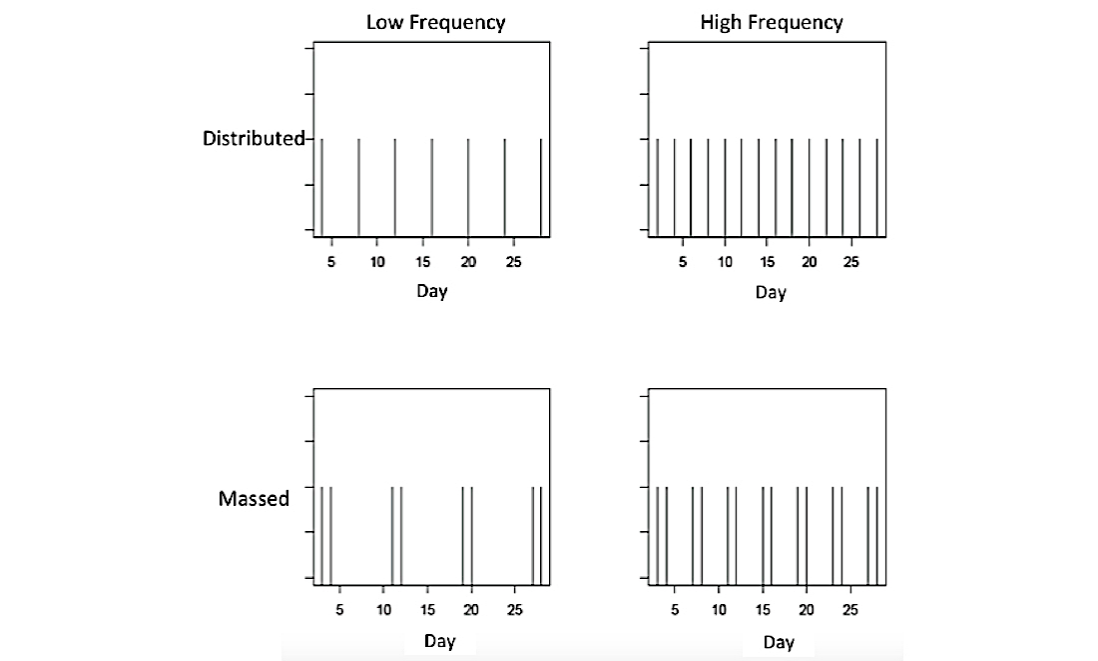
Reminder schedules used in the experiment.

**Figure 3:**
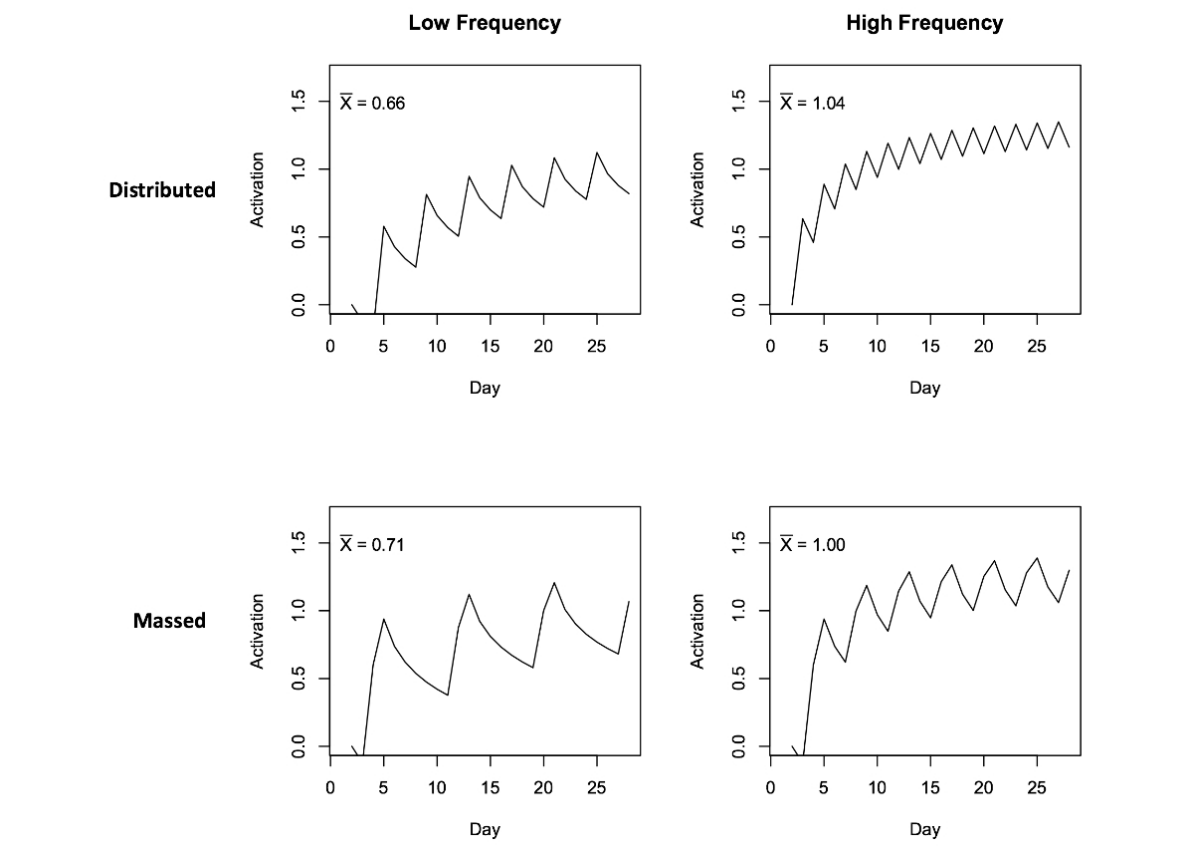
Simulated base-level learning of implementation intentions as a function of different reminder schedules.

Figure 2 presents the reminding schedules used in our 28-day study. The experiment manipulated the total frequency of reminders (7 or 14 reminders over 28 days) and the distribution of presentation (massed or distributed). Each vertical bar in Figure 2 indicates the day on which a reminder was sent to our participants. In the massed conditions, some reminders occur with less temporal spacing. The base-level learning parameters used to plot the base-level activation in Figure 3 are from Pavlik and Anderson [37], and they illustrate the practice, forgetting, and spacing phenomena: reminders are expected to boost up the base-level activation, but the activation decays without further practice, and distributed reminders are forgotten less quickly.

Each plot in Figure 3 also presents the predicted mean activation level of the implementation intention over the full 28 days for each condition (upper left corner of each plot). Note that at low frequency of reminders, the mean activation level in the massed condition is greater than that of the distributed condition, but at high frequency, the mean activation of the distributed condition is greater than the massed condition. Thus, there is a predicted interaction of reminder distribution (massed, distributed) by frequency (low, high) and specifically, the average activation levels for the implementation interventions are predicted to be high frequency-distributed > high frequency-massed > low frequency-massed > low frequency-distributed. Behavior-change data from the mHealth experiment will be used to test for this predicted interaction and the specific ordering of success rates predicted by the model.

**Figure 4:**
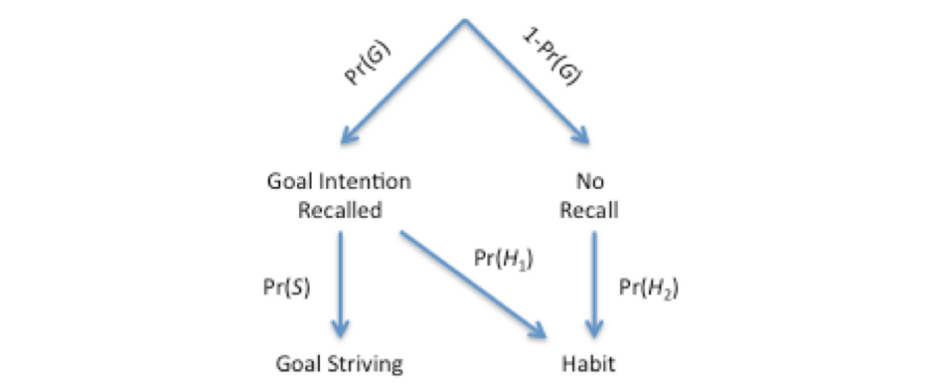
Probability tree for cognitive states and processes in the model.

### A Dynamical Model of Reminding and Habit Learning Effects for Each Individual

As discussed previously, theory suggests that behavior change involves dual systems: a goal-striving system that is heavily dependent on declarative memory and a habit-forming system that acquires and reinforces more automatic procedures for performance. Figure 4 captures the dual-system model that was fit to the data from the mHealth experiment. The model presented here is consistent with the ACT-R model presented above but is also similar to that of Pavel et al [45]. It is a dual-system model that includes (1) a habit or reinforcement learning system and (2) a declarative memory system with base-level learning of memory activation. The declarative memory model includes the Pavlik and Anderson [37] mechanisms to account for effects that may occur when remindings happen at variable spacing, which were manipulated in the experiment. If a reminder happens, there is an increase in the activation of the intention memory. If a goal success happens, there is habit reinforcement, as well as a strengthening of the activation of declarative memories for goal intentions and implementation intention memories.

**Figure 5:**
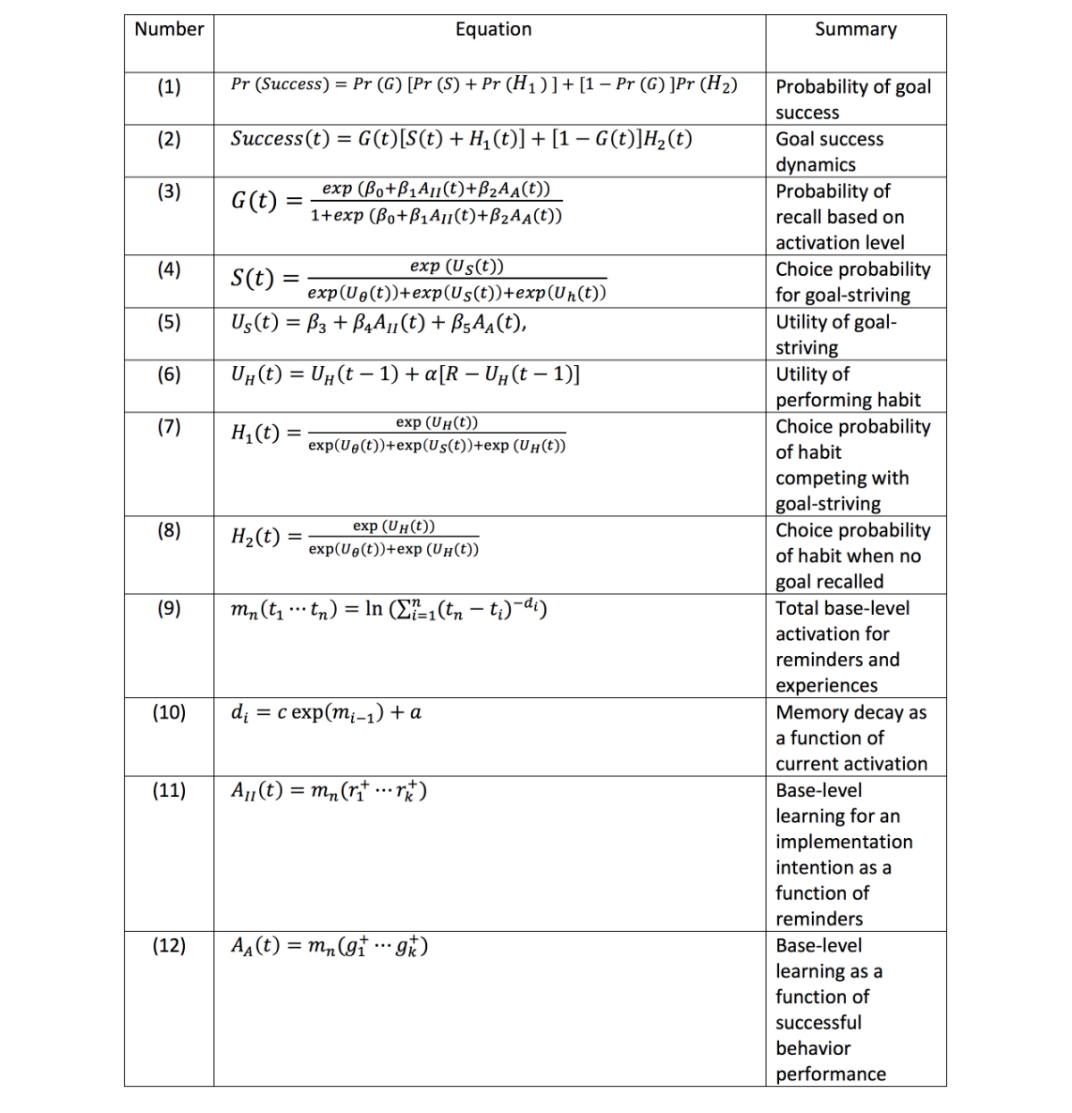
The adaptive control of thought-rational (ACT-R) dual-system model.

Figure 5 presents the details of the model in terms of 12 equations. On a given day, in the appropriate context for doing their goal behavior, a participant may recall their goal intention or not with probability Pr(*G*). If the goal intention is recalled, there are two routes to successful behavior: the goal behavior may be achieved through effortful goal striving with probability Pr(*S*), or if a habit has formed, it can be achieved by the new habit routines with probability Pr(*H*_1_). At first, goal-striving will be more probable, and after many repetitions, the habitual behavior will be more probable. After the habit is well practiced, even if the goal intention is not explicitly recalled, it may be executed with probability Pr(*H*_2_).

Altogether, the probability of participant success on a given day will be given by equation 1 (Figure 5). We can rewrite this as a dynamical equation dependent on day *t* as equation 2 (Figure 5). Where *Success* (*t*) is the probability of success of performing the goal, *G* (*t*) is the probability of remembering the goal intention, *H*_1_(*t*) is the probability of a habit routine given the goal intention has been recalled, and *H*_2_(*t*) is the probability of the habit given no goal intention recall.

The probability of recall based on activation level is given in equation 3 (Figure 5), where *A*_II_ is the base-level activation from reminders, and *A*_A_ is the base level activation from past experiences of actually doing the behavior (declarative memory of the experiences), and *β*_0_, *β*_1_, and *β*_2_, are scaling and slope parameters to be estimated.

As shown in Figure 1, ACT-R chooses productions based on the utility learned for those productions. The choice of goal-striving production versus habit productions is given by Equation 4 (Figure 5), where *U*_θ_ is a threshold utility that essentially captures the other behaviors competing with (or impeding) the choice of the goal behaviors. *U*_s_ is the utility of the goal-striving productions that we assume is also dependent on the activation strength of the implementation intention in memory as given in equation 5 (Figure 5).

The habits associated with the goal behavior are learned according to the utility learning equation 6 (Figure 5), where *R* is the reward value associated with successfully performing the targeted goal behavior. The performance of a habit competing with goal-striving is given by the probability *H*_1_*(t)* in equation 7 (Figure 5). In the case where the goal intention has not been recalled, but there is a habit that is being learned, the habit productions just compete with the background threshold, and the probability of the habit is *H*_2_*(t)* in equation 8 (Figure 5).

What remains to be defined is the declarative memory model that captures the base-level learning effects from the reminders and the successful behavior experience. As presented in Figure 1, each time, *t*_i_, a reminder, or experience happens, there is an increment of activation that decays as a power function with decay parameter *d*_i_. The total base-level activation is just the log of the sum of all those decaying increments as given in equation 9 (Figure 5). One complexity is that the decay parameter on each activation increment for a reminder or experience can vary as a function of the current level of activation, as defined in equation 10 (Figure 5), where *c* and *a* are scaling and slope parameters. This fits the observation that forgetting is slower when interpractice time is longer (spaced). So the base-level learning for the implementation intention, in equation 11, is defined by the times at which the reminder happened (*r*_1_^+^*, … r*_k_^+^*)*, and the base-level activation because of successful behaviors given in equation 12 is defined by the times at which the experiences happened, (*g*_1_^+,^*…,g*_k_^+^*)*.

### Summary of Aims

A 28-day exploratory mHealth experiment was conducted to investigate ACT-R predictions about the effects on achievement of behavior-change goals of implementation intention reminders and of prior goal achievement. The experiment uses an mHealth app that engages users to select goals to do new healthy behaviors (eg, eating more vegetables, eating more slowly, and increased walking time per day) to be performed every day and to set implementation intentions to do those behaviors.

The aims of this study were to

- Perform exploratory data analyses for signature phenomena predicted by the ACT-R theory. Memory for implementation intentions is predicted to (1) improve with the cumulative frequency of reminders and cumulative frequency of performance of the goal behaviors (practice effects) and (2) diminish with the time since presentation of past reminders and time since past goal performances (forgetting effects).
- Test-specific ACT-R predictions about the effects of reminder schedules on memory for implementation intentions, as revealed in participants’ rates of daily adherence to behavior-change goals (summarized in Figures 2 and 3)
- Model each individual’s daily goal achievement data using a dynamical mathematical model based on ACT-R. The ACT-R model captures the mechanisms underlying the role of reminders in amplifying the effects of implementation intentions during the volitional goal-striving phase of behavior change, as well as the gradual learning of new habits.

## Methods

We first present the mHealth experiment and then summarize the analysis methods that were applied to explore the ACT-R model of implementation intentions, remindings, and habit formation.

### Experiment

#### Design

An incomplete factorial design was used in this experiment (see Table 1). All participants were asked to choose a healthy behavior goal and to set implementation intentions. Participants were stratified by *self-efficacy*: their confidence to complete the selected behavior goal. Participants were assigned to one of two *reminder* conditions: presented versus absent. Self-efficacy and reminder conditions were crossed. Nested within the reminders presented condition was a 2 X 2 crossing of *frequency* of reminders sent (high, low) by *distribution* of reminders sent (distributed, massed). Participants in the low frequency condition were sent a total of 7 reminders, and participants in the high frequency condition were sent 14 reminders. Participants in the distributed conditions were presented reminders at uniform intervals: high frequency distributed participants were sent reminders on days 2, 4, 8, 10, 12, 14, 16, 18, 20, 22, 24, 26, and 28, whereas low frequency distributed participants were sent reminders on days 4, 8, 12, 16, 20, 24, and 28. Participants in the massed distribution conditions were sent reminders in clusters (lower row of plots in Figure 2): high frequency massed participants were sent reminders on days 3, 4, 7, 8, 11, 12, 15, 16, 19, 20, 23, 24, 27, and 28 and low frequency massed on days 3, 4, 11, 12, 19, 20, 27, and 28. In total, there were 10 conditions: 2 (self-efficacy) X 2 (frequency) X 2 (distribution)=8 in the reminder-present condition, plus 2 (self-efficacy) in the reminder-absent condition.

#### Participants

N=64 participants were recruited using the email list of a large university, Craigslist, and Nextdoor. Participants were paid US $50 in gift cards for their participations. Participants ranged in age from 25 to 71 years, with a median of 30 years. Internet protocol addresses suggested that participants came from eight different US states, and one came from India, with the majority coming from California and Michigan. Participants were randomly assigned to the 10 cells of the incomplete factorial design, resulting in slightly unbalanced cells with 5 to 8 participants per cell (Table 1). Pooling across the cells in Table 1, there were N=34 participants in the low self-efficacy conditions and N=30 in the high self-efficacy conditions. Pooling across reminders conditions, there were N=51 participants in the reminders presented conditions and N=15 in the reminders absent conditions. There were N=23 low frequency and N=28 high frequency participants and N=26 distributed distribution and N=25 massed distribution participants.

#### Materials

Four types of health behavior goals (habits) were developed by the nutrition and exercise specialist on our team: eating slowly (12 habits), walking (19 habits), food journaling (11 habits), and eating vegetables (6 habits). N=1500 participants from Mechanical Turk rated subsets of these habits on habit *difficulty* and perceived *self-efficacy*. The difficulty question was “ *how difficult is it for you to complete this goal everyday for the next 7 days?:* 1-10 scale,” and the self-efficacy question was “ *how confident are you that you can complete this goal everyday for the next 7 days?: 0%-100% scale.* ” Reported difficulty and self-efficacy rating were highly negatively correlated. Food journaling tasks did not show much variability in difficulty ratings and were dropped from the study. For the remaining three habits: eating slowly, walking, and eating vegetables, we selected the three most difficult habits and the three easiest habits. An example habit was “Stretch for 10 minutes and walk for 30 minutes in the afternoon.” An example of an implementation intention reminder for this habit was “Remember to stretch for 10 minutes and walk for 30 minutes in the afternoon—at in my neighborhood with a friend.” The complete list of habits is in Multimedia Appendix 1.

#### PARC Coach

PARC Coach is a mobile app developed to study behavior change interventions in an mHealth setting. It implements only the most central features to reporting behavior and delivering interventions. PARC Coach has a reporting home page on which people report whether they have met their daily goal. Informational pages were available for every behavioral goal.

#### Procedure

Upon creating an account and first time logging in, they were asked to select a class of habit to pursue: eat slowly, walking, or eating more vegetables. Upon habit selection, participants were randomly assigned to either a high self-efficacy or low self-efficacy goal for that habit class. Upon being assigned the specific habit, participants were asked to rate their self-efficacy for achieving the assigned goal with the question “*how difficult is it for you to complete this goal everyday for the next 7 days?:* 1-10 scale.”

Self-reported daily goal achievement reports were collected in the PARC Coach app. The app contains a reporting page in which participants click a button to indicate whether they did their goal behavior.

Participants were then asked to set an implementation intention with the following components (possible responses in parentheses): (1) *Which part of day will you do practice this habit?* (morning, afternoon, evening) or *which meal will you like to try this at?* (breakfast, lunch, or dinner), (2) *Where will you do this activity?,* (3) *Who will you do this activity with?*, and (d) *When will you do this activity?* (event time).

If participants were in a reminder condition, they were additionally asked *How long before the event would you like to be reminded of your task?* (reminder duration). In the reminder condition, the participants’ selection of an event time and a reminder duration were combined with the reminder schedule to compute when to send the reminder. 

**Table 1:** Number of participants assigned to cells of the incomplete factorial design.

Self-efficacy	Reminders	Frequency	Distribution	Participants (n)
Low	Presented	Low	Distributed	6
Low	Presented	Low	Massed	6
Low	Presented	High	Distributed	7
Low	Presented	High	Massed	8
Low	Absent			7
High	Presented	Low	Distributed	6
High	Presented	Low	Massed	5
High	Presented	High	Distributed	7
High	Presented	High	Massed	6
High	Absent			6

Reminders were sent by SMS to the participants’ mobile phone. The content of the reminders was determined by the habit and the participants’ implementation intentions. Participants were expected to acknowledge reminders by clicking “OK.” The reminder was canceled if it wasn’t acknowledged until the event time. The sending of reminders and the acknowledgment of their receipt was automatically logged by the PARC Coach app.

### Analyses

#### Exploratory Data Analyses for Signature Memory Phenomena

ACT-R predicts that the effects of reminders on the memory activation levels of implementation intentions will exhibit practice and forgetting effects. Memory activation is predicted to increase with the frequency with which reminders are processed (practice) and decrease as with decreases in the recency since reminders were processed (forgetting). When a reminder is sent to participants, there is a chance that they may ignore the reminder. When participants acknowledged the receipt of reminders, we assume that is an indicator that they actually attended to and processed the reminder.

We performed a set of exploratory analyses on the basic relationship of reminder schedules to achieving behavior-change goals (adherence). For every participant, on every day, the dependent variable of goal adherence was coded (success=1, failure=0). For every participant, on every day, we also coded how many times a reminder had been acknowledged as received since the start of the experiment (*frequency acknowledged*) and how many days since the last reminder had been acknowledged (*recency acknowledged*); how many time a reminder has been sent since the beginning of the experiment (*frequency sent*) and how may days since the last reminder had been sent (*recency sent*); and how many days adherence had been reported in the past (*frequency adherence*) and how may days since the last report of success (*recency adherence*).

We, for each type of input variable (reminder acknowledged, reminder sent, past goal adherence) at every level of frequency and recency, computed the mean probability of adherence (ranging from 0-1) and computed simple linear regressions of the form

Adherence ~ β_F0_+ β_F1*_ Frequency,

and

Adherence ~ β_R0_+β_R1*_ Recency,

and computed the goodness-of-fit R^2^ statistics. In addition, we analyzed the contribution of self-efficacy and reminder frequency and recency with a logistic regression:

logit(Adherence) ~ β_0i_ + β_0_ + β_1_ S + β_3_ R

where *S*=self-efficacy (a categorical variable), *F*=frequency acknowledged, *R*=recency acknowledged, and is a random coefficient estimated for each participant *i*.

#### Analysis of Specific ACT-R Predictions About Reminder Schedules

We performed a logistic regression analysis of goal adherence data within the 2 (frequency) X 2 (distribution) factorial conditions in which reminders were presented. This analysis serves as a test for the specific pattern of a priori ACT-R predictions about the effects of reminder schedules on memory for implementation intentions. Those predictions are summarized in Figures 2 and 3. Specifically, based on the mean base-level activations, ACT-R predicts an interaction of frequency X distribution with the average activation levels for the implementation interventions predicted to be high frequency-distributed > high frequency-massed > low frequency-massed > low frequency-distributed.

#### Fit of the ACT-R Dual-System Mathematical Model to Individual-Level Data

A fit of the ACT-R mathematical model (defined in equations 1-12) to the data for every individual and every day was obtained by minimizing the Brier score between model-predicted probability success and observed success using the *R* optimx package using a quasi-Newton method called limited-memory BFSG, which allows one to bound the parameter search (by providing upper and lower boundaries).

## Results

### Signature Practice and Forgetting Phenomena Because of Reminders and Performance

**Figure 6:**
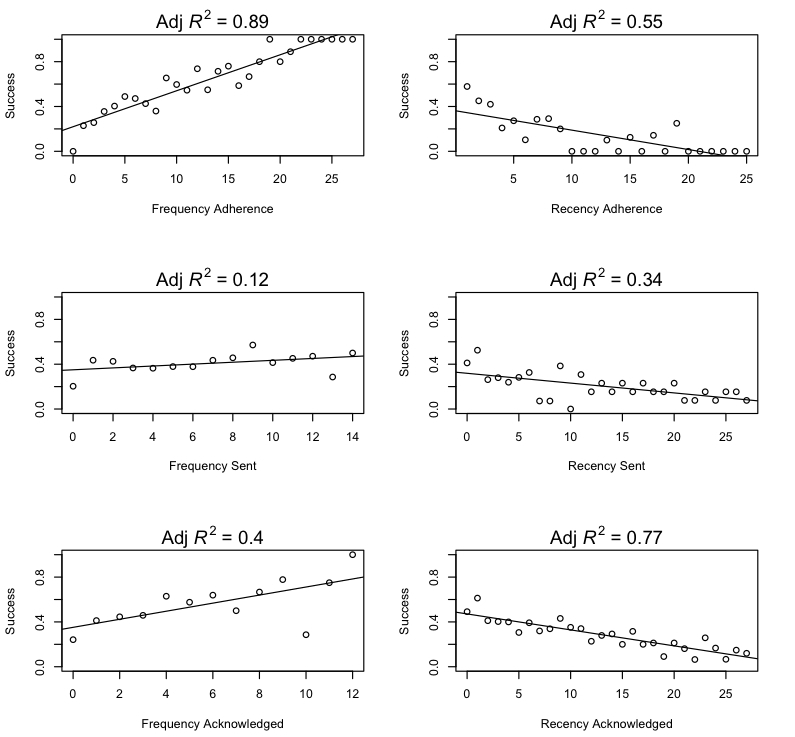
Factors related to the likelihood of a participant succeeding at behavioral goal on a given day. Frequency of adherence is the cumulative number of past “success report” days. Recency of adherence is the number of days since last “success report.” Frequency sent is the cumulative count of reminders previously sent. Recency sent is the number of days since the last reminder was sent. Frequency acknowledged is the cumulative count of previously acknowledged reminders. Recency acknowledged is the numbers of days since the last acknowledgement of a reminder. Adjusted R^2^ values are based on linear regressions.

Practice and forgetting effects are key signatures of declarative and procedural memory [46,47]: improvements generally accrue with repeated practice or remindings and decay over time without continued practice or reminding. Figure 6 demonstrates that these signature phenomena are apparent in the success rates of the participants. Each point in the plots is the mean observed probability of participants reporting success at achieving a goal on a given day as a function of different frequency and recency factors. The top two plots present the probability of reported success as a function of frequency and recency of past goal success. The middle two plots present the observed probability of success as a function of the frequency and recency of sent reminders. The bottom two plots present the observed probability of success as a function of frequency and recency of acknowledging the sent reminders. Each plot also presents a best-fit linear regression line, as well as the adjusted R^2^ for the regression.

In general, the frequency and recency of past success (adherence) tends to show medium-to-strong relationships to current success. The frequency and recency of acknowledged reminders tends to show stronger effects than the frequency and recency with which those reminders were sent. This is unsurprising, as one would expect that sent reminders could be ignored, but acknowledgment indicates that the participant actually processed the reminder.

Table 2 presents a logistic regression, with the daily success or failure as the response variable, participants as a random effect, and the predictors being the self-efficacy factor and the observed frequency and recency of reminder acknowledgment. Self-efficacy was not significant, frequency acknowledged was marginally significant, and the recency acknowledged was highly significant. 

**Table 2:** Logistic regression of daily success in achieving self-selected goals on self-efficacy and frequency and recency of acknowledged implementation intention reminders.

Predictor	Coefficient (standard error)	Odds ratio (95% CI)	*P* value
Intercept	−0.5696 (0.3185)	0.5657 (−1.2231 to 0.0602)	.007
Low self-efficacy	−0.1197 (0.4180)	0.8872 (−0.9655 to 0.7232)	.77
Frequency acknowledged	0.0694 (0.0410)	1.0717 (−0.0116 to 0.1505)	.09
Recency acknowledged	−0.0490 (0.0104)	0.9522 (−0.0700 to −0.2852)	<.001

An analysis of deviance also showed that including frequency acknowledged to the base model of recency acknowledged was marginally significant χ^2^_1_=2.8 *P*=.09, and adding self-efficacy to the acknowledgment factors was not, χ^2^_1_=0.1.

### Effects of Implementation Intention Reminder Schedules on Behavior Success: A Test of ACT-R Memory Predictions

Table 3 shows the mean proportion of days on which participants reported succeeding at their behavior change goals. Notably, the condition in which participants received reminders was significantly better than the conditions in which others did not receive reminders, (logistic regression on reminders vs no reminders: coefficient=2.018, SE=0.572, OR=7.52, 95% CI 0.9037-3.2594, *P*<.001).

The pattern of success rates in Table 3 suggests an interaction of reminder frequency by distribution, as predicted by the ACT-R base-level learning theory (Figure 3). A logistic regression was performed on the average rate of participant success in performing their goals over 28 days in the 2 X 2 factorial conditions of distribution (distributed, massed) X frequency (low, high). The results of this regression are presented in Table 4. Although there are no main effects of the frequency or distribution variables, there was a highly significant interaction. An analysis of deviance showed that the model with the interaction term was significantly better than a reduced model without the interaction term, χ^2^_1_=13.056, *P*=.0003. Post hoc linear contrasts show that the high frequency-distributed condition produced higher participant success than the high frequency-massed, *z*=4.441, SE=0.2853, *P*<.001; the high frequency-massed produced higher success than the low frequency-massed, *z*=3.041, SE=0.2609, *P*=.003; and the low frequency-massed produced marginally higher success than the low frequency-distributed, *z*=1.929, SE=0.2663, *P*=.069.

Table 1 shows that each of the 2 X 2 cells of the frequency X distribution factorial have N=11 to N=14 participants. Gelman and Carlin [48] suggest that such small-sample experiments warrant an analysis of two kinds of potential experimental design errors: (1) the probability that the estimate of the effects is in the wrong direction (*Type S [sign] error*) and (2) the factor by which the magnitude of an effect might be overestimated (*Type M [magnitude] error*). We followed the procedure recommended by Gelman and Carlin. First, we went to the literature to identify a possible range of true effects sizes for our experiment. A recent meta-analysis [49] of the effect size (ES) of behavior-change interventions [50] indicates an ES=0.37 in improving physical activity and diet over the short term (<12 weeks), with a 95% CI 0.26-0.48. Next, using the *retrodesign()* function [48] we determined the *power*, *Type S*, and *Type M* error for the interaction effect in Table 4. At ES=0.37, the probability that a replication would be statistically significant at alpha=.05 was *power*=0.39, the probability that a replicated estimate would have the incorrect sign was *Type S error*=0.0003, and the expectation of the ratio of the estimated interaction effect to a true ES=0.37 was *Type M error*=1.56. It is unlikely that the sign of the significant interaction is incorrect (*Type S error*), and the magnitude of the interaction in Table 4 is likely overestimated by a factor of 1.56 (*Type M error*). For ES=0.26: *power*=0.22, *Type S error*=0.0038, *Type M error*=2.13; for ES=0.48: *power*=0.59, *Type S error*<0.0001, *Type M error*=1.30.

These analyses suggest that the pattern of participant success at their behavior-change goals over 28 days is consistent with the ACT-R theoretical predictions of how the variations in reminder schedules affect the base-level activation of participants’ implementation intentions in declarative memory.

### Fit of the ACT-R Dual-System Mathematical Model to Individual-Level Data

**Figure 7:**
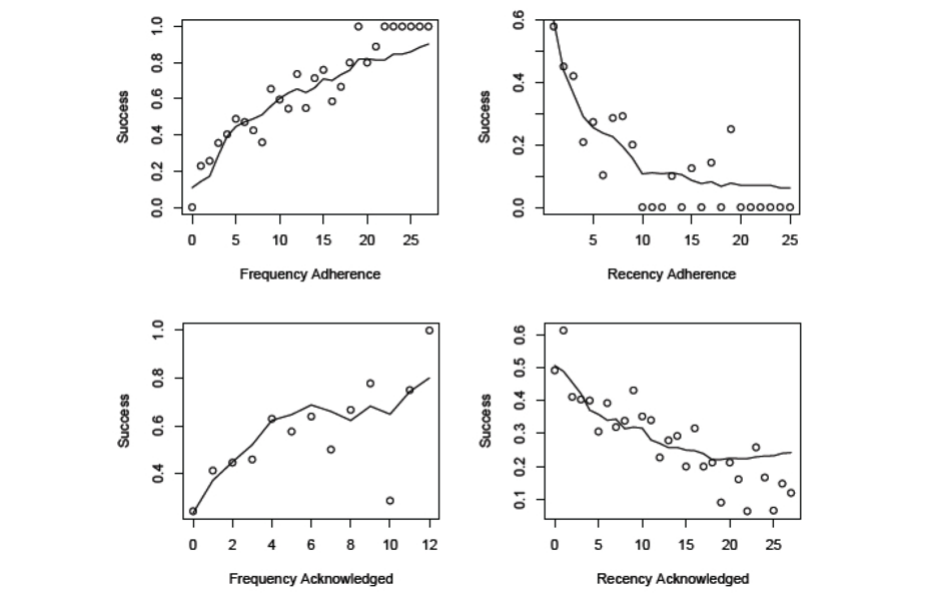
Fit of the adaptive control of thought-rational (ACT-R) dual-system model to daily success in performing behavior goals.

Figure 7 plots the goal success predictions of the dynamical model against the observed data as functions of past adherence (frequency and recency) and reminders acknowledged (frequency and recency). The points are the observed probabilities of success, and the lines are the model predicted probabilities. Each point is the mean of the observed individual daily success for participants at a given level of recency or frequency, and similarly, the lines are the means of the model’s predictions for each individual on each day, pooled by level of frequency and recency. Parameter estimates for the fitted more are presented in Table 5. The Brier score on this fit was 0.1724.

Figure 7 suggests that the model is doing a reasonable job of predicting the observed frequency and recency effects because of the reminding interventions, as well as the recency and frequency effects because of the practice of the target behavior. The model does appear to predict a more sublinear relationship between the recency of reminder acknowledgment than is present in the observed data.

The parameters estimated in Table 5 also appear to be generally reasonable. In predicting the probability of recalling a goal and striving to do a new behavior, the activation levels of implementation intentions and memory for past goal performance are both associated with nonzero positive weights (*β*_1_ and *β*_2_). Similarly, the base-level activation of implementation intentions and memory for past goal performance are also positively weighted in determining the utility of goal-striving performance (*β*_4_ and *β*_5_). The values of parameters, *a*, *c*, determining the base-level learning that we estimated are different than those found in controlled laboratory studies [37], where alpha=.177 and *c*=0.217. It should be noted that the Pavlik and Anderson study involved multiple blocks of multiple trials within a study day, and the spacing of reminders was manipulated within a 60 to 90 min study session. In our study, we were manipulating the spacing of reminders over many days.

**Table 3:** Mean proportion of days on which participants reported success in achieving their behavior-change goal (standard deviation in parentheses).

Distribution	Frequency	
	Low	High
Distributed, mean (SD^a^)	0.32 (0.33)	0.55 (0.24)
Massed, mean (SD)	0.34 (0.22)	0.38 (0.26)
No reminders, mean (SD)	0.18 (0.23)	

^a^SD: standard deviation.

**Table 4:** Logistic regression on the average rate of participant success in performing their goals over 28 days in the 2 X 2 factorial conditions of distribution (distributed, massed) X frequency (low, high).

Predictor	Coefficient (standard error)	Odds ratio (95% CI)	*P* value
Intercept	−0.6305 (0.1197)	0.5323 (−0.8681 to 0.3984)	<.001
High frequency	0.1630 (0.1584)	1.1770 (−0.1467 to 0.4746)	.30
Distributed distribution	−0.1167 (0.1672)	1.1238 (−0.4448 to 0.2112)	.49
High frequency X distributed	0.7994 (0.2215)	2.2242 (0.3656-1.2341)	<.001

**Table 5:** Parameter estimates for the adaptive control of thought-rational (ACT-R) dual-system model.

Parameter	Value	Description
*β*_0_	8.107708	Scaling parameter on activation for predicting goal recall
*β*_1_	4.896597	Weight, implementation intention activation in predicting probability goal recall
*β*_2_	3.535064	Weight, memory activation of performing goals in predicting probability goal recall
*β*_3_	−0.732805	Scaling parameter on utility of goal striving productions
*β*_4_	0.297554	Weight, implementation intention activation in utility of goal striving productions
*β*_5_	1.396243	Weight, memory activation of performing goals in utility of goal striving productions
*a*	1.000000	Scaling parameter on base-level activation learning
*c*	0.077193	Slope parameter on base-level activation learning
*U*_0_	−3.818326	Initial utility of new habit
*α*	0.291842	Utility learning rate for the habit
*R*	0.000000	Reward value for new habit

## Discussion

Consistent with previous research on implementation intentions [22], we found that reminders of implementation intentions sent as SMS text messages have a boosting effect on success in achieving behavior change goals. Further exploratory analysis suggested that the effects of reminding on behavioral success appear to show signatures long associated with human memory: a decay of effectiveness because of forgetting and improvement with repetition. The analysis also indicates improvement with behavior practice, which is ubiquitous in the procedural learning of skills and habits. A mathematical model based on ACT-R captures the dynamics of people accomplishing their goal behaviors under the influence of implementation intentions, reminders, and their own past performance.

For practitioners, prior research had suggested the utility of using implementation intentions in mHealth [22,51]. Our results suggest that reminders can boost the effectiveness of implementation intentions in ways predicted from basic memory theory. Our results also suggest the importance of the recency factor: the number of days since a reminder was acknowledged accounts for a large proportion of the variance in goal adherence (Figure 6) and was highly significant in the analysis reported in Table 2. Attending to reminders is associated with performing the behavior soon thereafter. This might suggest that mHealth reminders be triggered by some combination of nonadherence and time since the last reminder was sent. One concern might be that sending more reminders might just cause users to decide to ignore them. As a post hoc analysis, we examined whether the acknowledgment of reminders was reduced when reminders were temporally clustered in the massed condition, in contrast to the distributed condition. We found no statistical difference, but we still were not sending reminders more than once a day.

It has been argued [52] that the current menagerie of behavior change theories [11,50] needs to be refined or replaced with precise models that yield predictions at the granularity of assessments and interventions that are delivered by mHealth systems in the ecology of everyday life. Such models would provide a rigorous foundation for engineering sophisticated, individualized interventions that are optimally delivered in the right contexts at the right time. In the present research, we worked from the existing ACT-R theory [7] to propose a model of goal-striving and habit formation that would predict the effects of implementation intentions and reminders. Previous research [18] had suggested that human declarative memory mechanisms were implicated in the effectiveness of this EBI. ACT-R predicts the dynamics of declarative memory retrieval in response to additional training or use, and so the theory was extended to make predictions about the dynamics of implementation intention effectiveness as a function of the timing of reminder interventions.

Related ACT-R models [34] have been developed to predict the dynamics of self-efficacy and goal success in users of an mHealth app called DStress [10]. In that case, there were two kinds of modeling: (1) full simulations in the ACT-R cognitive architecture and (2) dynamical mathematical models approximating the ACT-R mechanisms, similar to the approach presented here. The ACT-R–inspired mathematical models are also similar to the dynamical models presented in Pavel et al [45], which also used a dual-systems approach for goal-striving and habit formation. In other applied domains such as cognitive tutoring [53] and language learning [36], it has been useful to develop user models that are approximations to the detailed ACT-R simulation architecture, yet, still support prediction by computation (see also, [54]).

To repeat an argument made by Pirolli [34], the motivation for developing mHealth theories by extending theories of the human cognitive architectures rests on four theses [55,56]: (1) the *integration thesis*, that cognitive architectures provide a unified account of how the modules of the mind function together to produce coherent behavior and can provide a basis for an integration across specialized domains of EBIs, theories of behavior change, and multiple systems and mechanisms of action in behavior change; (2) the *decomposition thesis*, that long-term behavior change can be decomposed to learning and intervention events occurring at a much finer granularity of time; (3) the *modeling thesis,* that models in cognitive architectures can provide a basis for bridging those events at the small scale to the dynamics of behavior change occurring at the large scale; and (4) the *relevance thesis*, that long-term changes and outcomes can be improved by modeling and predicting specific just-in-time interventions in contexts that are occurring at the smaller time scales in the ecology of the everyday lives of people wishing to change.

## Appendix

Multimedia Appendix 1 Habits used in the Implementation Intention Study.

## Abbreviations

ACT-R: adaptive control of thought-rational
EBI: evidence-based interventions
mHealth: mobile health
OR: odds ratio
SE: standard error
TPB: theory of planned behavior
