# Supplementary material for: Implementation Intention and Reminder Effects on Behavior Change in a Mobile Health System: A Predictive Cognitive Model
Source: J Med Internet Res. 2017 Nov 30;19(11):e397. doi: 10.2196/jmir.8217 (PMC5730820; doi:10.2196/jmir.8217)
Supplement: Multimedia Appendix 1 [file jmir_v19i11e397_app1.pdf]

Habits used in the Implementation Intention Study

Eat slowly

1. Put your utensils down between bites and do not pick them up again until you have chewed your food completely and swallowed it

2. Take a deep breath between bites

3. Chew your food until it liquefies in your mouth

4. Chew each bite of food 10 times

5. Chew each bite of food 15 times

6. Chew each bite of food 20 times

7. Add extra 5 minutes to eat your meal

8. Add extra 10 minutes to eat your meal

9. Take smaller bites

10. Cut down on any other activity while eating

11. Take 30 minutes to complete your meal

12. Take 45 minutes to complete your meal

Keeping a food journal

1. Text what you ate to us

2. Text what you ate and your mood to us

3. Text what you ate and how fast you ate

4. Text what you ate and why you made the choice

5. Text what you ate, mood, how fast you ate

6. Text what you ate, how fast you ate, why you made the choice

7. Text what you ate, how fast you ate, why you made the choice, how fast you ate

8. Take a picture of your meal and send it to us

9. Count the calories using an app and text it to us
10. Take a picture of your meal, count calories, and send those to us
11. Take a picture of your meal, count calories, notice mood, and send those to us
12. Take a picture of your meal, count calories, notice mood and the reason why you ate it and send those to us

Walking (forcing people to do this might hurt them?)

1. walk 10 minutes
2. stretch for 5 minutes and walk for 10
3. walk 15 minutes
4. stretch for 5 minutes and walk for 15
5. walk 20 minutes
6. stretch for 5 minutes and walk for 20
7. walk 25 minutes
8. stretch for 10 minutes and walk for 25
9. walk 30 minutes
10. stretch for 10 minutes and walk for 30
11. walk 35 minutes
12. stretch for 10 minutes and walk for 35
13. walk 40 minutes
14. stretch for 10 minutes and walk for 40
15. walk 45 minutes
16. stretch for 10 minutes and walk for 45

Stretching

- Stretch for 5 minutes

- Stretch for 10 minutes
- Stretch for 15 minutes

Eat veggies

1. Record the veggies you consume in a meal and send those to us
2. add 1 serving of a vegetable to a meal
3. Add 2 servings of vegetables to a meal
4. have a salad for a meal
5. double the serving of your favorite veggie to a meal
6. eat a new veggie
